# A Comprehensive Scientometric Analysis of Placebo and Nocebo Effects in Pain Research: From Placebo Mechanisms to Clinical Trials

**DOI:** 10.1111/jep.70570

**Published:** 2026-09-13

**Authors:** Francesca Borghesi, Valeria Volpino, Elisa Rho, Giorgia Cecconato, Francesco Campaci, Pietro Cipresso, Alessandro Piedimonte, Elisa Carlino

**Affiliations:** ^1^ Department of Psychology University of Turin Turin Italy; ^2^ “Rita Levi Montalcini” Department of Neurosciences University of Turin Turin Italy; ^3^ Carlo Molo Foundation Turin Italy; ^4^ Istituto Auxologico Italiano IRCCS, U.O. di Neurologia e Neuroriabilitazione Milan Italy

**Keywords:** bibliometric mapping, pain research, placebo effect, placebo mechanism, scientometric analysis

## Abstract

Placebo and nocebo phenomena are central in neuroscience, psychology, and clinical research, yet comprehensive bibliometric analyses remain limited. This study mapped placebo and nocebo research in pain, distinguishing mechanistic studies from randomised controlled trials (RCTs), using data from the Web of Science Core Collection up to March 2025, which yielded 1246 mechanistic studies and 35,134 RCTs. Analyses performed with the Bibliometrix R package included descriptive statistics, co‐citation and keyword co‐occurrence networks, and local citations to assess internal impact. Mechanistic studies grew 3.97% annually, focusing on expectation, conditioning, and pain modulation, with key contributions from Benedetti, Colloca, and Wager, concentrated in the USA, Germany, Italy, and the UK. RCTs grew 3.52% per year, emphasising systematic reviews and methodological standards (Jadad scale, PRISMA), led by Moore and Manchikanti, with the USA, China, and the UK dominating output. Co‐citation analyses identified mechanistic clusters around opioidergic and dopaminergic pathways and prefrontal–cingulate circuits, and RCT clusters emphasising methodological rigour and pain outcomes. Keyword analyses highlighted pain, analgesia, and expectation as central themes, with emerging trends reflecting evolving clinical applications and innovative trial designs. This scientometric mapping underscores the dual role of placebo as a neurobiological phenomenon and methodological cornerstone, offering a comprehensive overview of the evolution, structure, and thematic organisation of placebo and nocebo research in pain.

**Abbreviations**
A& HCI: Arts and Humanities Citation Index
BKCI‐S: Book Citation Index–Science
BKCI‐SSH: Book Citation Index–Social Sciences & Humanities
CPCI‐S: Conference Proceedings Citation Index–Science
CPCI‐SSH: Conference Proceedings Citation Index–Social Sciences & Humanities
CPI: Citation index
ESCI: Emerging Sources Citation Index
IC: Index Chemicus
PRISMA: Preferred Reporting Items for Systematic Reviews and Meta‐Analyses
RCT: Randomised Controlled Trial
SCI‐EXPANDED: Science Citation Index Expanded
SSCI: Social Sciences Citation Index
WoS: Web of Science

## 1 Introduction

From its first appearance in medical literature in the seventeenth century, the concept of ‘placebo’, and later its opposite ‘nocebo’, has undergone multiple semantic and conceptual shifts [1, 2, 3, 4, 5, 6]. This led to significant confusion in the use of the terms ‘placebo/nocebo effects’, ‘placebo/nocebo responses’ and their broader interpretation as ‘placebo/nocebo’ itself [7, 8, 9]. Today, placebos are widely used as a control condition in Randomised Clinical Trials (RCTs) [10, 11, 12], but also play a central role in experimental research investigating their psychological and neurobiological mechanisms [13, 14].

Despite the growing interest in these phenomena, only two bibliometric analyses have been conducted to identify publications dealing with the placebo/nocebo phenomena [15, 16], while different narrative reviews have been published defining the terms and describing the history of placebos and nocebos [1]. Even if narrative reviews offer qualitative insights and interpretations of a topic, they do not provide a quantitative analysis of these insights. On the contrary, scientometric analyses aim to quantitatively measure and evaluate scientific research output, productivity, impact, and trends within a particular field. However, the two bibliometric analyses present in the literature were conducted on a small sample of data and investigated only studies focused on the placebo effect [15, 16], namely studies on the neurobiological and psychological mechanisms underlying the placebo phenomenon. Conducting a scientometric analysis that a priori focuses solely on these studies is undoubtedly valuable for understanding the phenomenon. However, a comprehensive understanding of how placebo and nocebo knowledge has evolved requires the exploration of both mechanistic studies, in which placebo and nocebo effects constitute the primary object of investigation, and randomised clinical trials, in which placebo serves primarily as a methodological tool and comparator condition. Distinguishing these two domains allows the characterisation of the dual role of placebo and nocebo in medical research, both as biopsychosocial phenomena and as fundamental components of clinical trial methodology.

Given the broad range of clinical applications of placebo and nocebo effects, the present study intentionally focuses on pain research. This choice was driven by methodological considerations, as restricting the analysis to a single clinical domain allowed us to obtain a more homogeneous dataset and perform a more robust and interpretable scientometric analysis. Furthermore, pain represents one of the most extensively investigated fields in placebo and nocebo research, both in terms of mechanistic investigations and randomised clinical trials [16, 17].

The present scientometric analysis aims to fill this gap by separately examining both placebo mechanistic studies and RCT placebo studies, to provide at the same time and with the same analysis techniques, a broad and unbiased overview of how research on placebo and nocebo in pain has evolved across both experimental and clinical landscapes. By systematically evaluating and synthesising existing literature, we seek to elucidate key trends, patterns, and developments in this field over time, to support researchers in orienting themselves within the complex landscape of placebo and nocebo research, enabling them to identify knowledge gaps, emerging trends, and evidence‐based directions for future studies and clinical implementation.

## 2 Literature Search Methods

### 2.1 Data Collection

The query ‘(placebo* OR nocebo*) AND pain* AND/NOT (“clinical trial*” OR “clinical‐trial*” OR “randomised trial*” OR “randomise trial*” OR “randomised‐trial*” OR “randomised‐trial*” OR “induction trial*” OR “induction‐trial*” OR “controlled clinical‐trial*” OR “controlled clinical trial*” OR “randomised‐controlled” OR “randomised controlled” OR “randomised‐controlled” OR “RCT*” OR “randomised double‐blind” OR “randomised double blind” OR “randomised double‐blind” OR “randomised double blind” OR “placebo‐controlled” OR “placebo controlled” OR “controlled trial*” OR “double‐blind” OR “double blind” OR “phase I” OR “phase II” OR “phase III” OR “phase IV” OR “study I” OR “study II” OR “study III” OR “study IV” OR “mg” OR “dose response*” OR “dose‐response*” OR “phase 1” OR “phase 2” OR “phase 3” OR “phase 4”)’ was used to recover data from Web of Science (WoS) Core Collection (Falagas et al., 2008), covering papers published until March, 2025. WoS core collection includes Citation Indexes, Science Citation Index Expanded (SCI‐EXPANDED)—1970‐present, Social Sciences Citation Index (SSCI)—1970‐present, Arts and Humanities Citation Index (A& HCI)—1975‐present, Conference Proceedings Citation Index‐ Science (CPCI‐S) –1990‐present, Conference Proceedings Citation Index‐ Social Science & Humanities (CPCI‐SSH)—1990‐present, Book Citation Index‐Science (BKCI‐S)—2009‐present, Book Citation Index‐Social Sciences & Humanities (BKCI‐SSH)—2009‐present, Emerging Sources Citation Index (ESCI)—2015‐present, Chemical Indexes, Current Chemical Reactions (CCR‐EXPANDED)—2009‐present (Includes Institut National de la Propriete Industrielle structure data back to 1840), Index Chemicus (IC)—2009‐present.

The query with *AND* was used to retrieve records for the *clinical trials* analysis, whereas the query with *NOT* was used to retrieve records for the *placebo mechanism* analysis.

### 2.2 Data Analysis

The initial searches yielded 32,948 records for the clinical trials dataset and 4,503 records for the placebo mechanisms dataset. During the subsequent screening, conducted independently and under blinded conditions by two reviewers (E.R. and G.C.), several records initially assigned to the placebo mechanisms dataset were found to meet the eligibility criteria for the clinical trials dataset and were therefore reclassified accordingly. Following this screening and reclassification process, as well as the exclusion of ineligible records, the final datasets comprised 35,134 records for the clinical trials analysis and 1,246 records for the placebo mechanisms analysis. All subsequent analyses were conducted on these final datasets. All the information on dataset screening and selection is summarised in the flowcharts provided as Supporting Materials (Supporting Information S1: Tables 1A,B). Several fields were included in the analyses: authors, title, keywords, keywords plus, references, and journals. RStudio (Bibliometrix package) [18] and Biblioshiny [19] were used for statistical analyses and visualisation of co‐citation networks [18]. The following analyses were conducted separately for the clinical trials and placebo mechanisms datasets. Data analysis for both scientometrics was divided into qualitative and quantitative analyses.

For the *qualitative analysis*, descriptive metrics were extracted to outline a preliminary framework, along with sources, publications, and authors' descriptive metrics. This analysis measures the impact and recognition of the research field of interest within the academic community by tracking publication trends, the most productive sources and authors, and the most cited and productive countries. Throughout our scientometric analyses, only *local citations* were considered in all citation counts, rather than all the existing citations a document received (i.e., only citations from other documents included in our selected dataset); this metric is used to assess the internal impact of the focus of interest.

For the *quantitative analysis*, network extraction was employed to inspect the structural and thematic organisation of the field. The structure of a network, which is broken down into clusters, consists of relationships between documents, keywords, or authors, contributing to the field's construction and development. Network analyses were based on *co‐citation (Walktrap clustering‐ repulsion 0.5, node* = *50)*, which refers to the frequency with which two references are cited together by subsequent articles and provides insights into the intellectual structure of the literature [20, 21, 22], authors' keyword *co‐occurrence (Placebo mechanism: Spin‐glass clustering, repulsion 0.5, node = 50; Clinical trials: Walktrap clustering, repulsion 0.5, node = 50)*, which clusters terms that frequently appear together in articles, revealing dominant themes and conceptual relationships, and *collaboration (Louvain clustering‐ repulsion 0.5, node* = *50)*, which maps co‐authorship relationships among authors, identifying collaborative communities and the social organisation of research within the field [23]. For each network, the clustering and association‐normalisation settings were selected after comparing alternative configurations and retaining those that produced the most cohesive and dense, interpretable network structure. For the authors' keyword co‐occurrence analysis of placebo mechanisms, we excluded terms that directly name the phenomenon— ‘placebo’, ‘placebo effect’, ‘placebo effects’, ‘placebo response’, ‘placebo responses’, ‘placebos’, and the corresponding ‘nocebo’ forms (‘nocebo’, ‘nocebo effect’, ‘nocebo effects’, ‘nocebo response’, ‘nocebo responses’, ‘nocebos’)—to better capture words associated with the broader concepts of placebo and pain.

Statistics used to describe the structural properties of co‐citation, co‐occurrence, and collaboration networks are *betweenness centrality*, *closeness centrality*, and *PageRank* [24]. Betweenness centrality values indicate the node's role in the information flow inside the network. A high betweenness assigned by the analysis to a document, a keyword, or an author depicts its significant role in the connections of its network. Closeness centrality measures the average ‘distance’ from one document, keyword, or author to all others, indicating the degree of information dissemination within the network. While betweenness centrality measures how much a node allows connections between other nodes in its network, closeness centrality measures how much a node is close (or interconnected) to the entire network. PageRank algorithm is used to determine the importance of a document, keyword, or author within a network, evaluating its relevance based on citations received from others. For instance, if we consider a single document from the selected dataset having high betweenness, closeness, and PageRank values, it means that the document functions as a bridge between different research clusters, its findings are highly shared by other documents, and it is cited by influential works. In our network analysis, the betweenness index resulted as the most informative one, while closeness indices and PageRank always show values around zero due to dense co‐citation networks and fragmentation of subfields.

**FIGURE 1:**
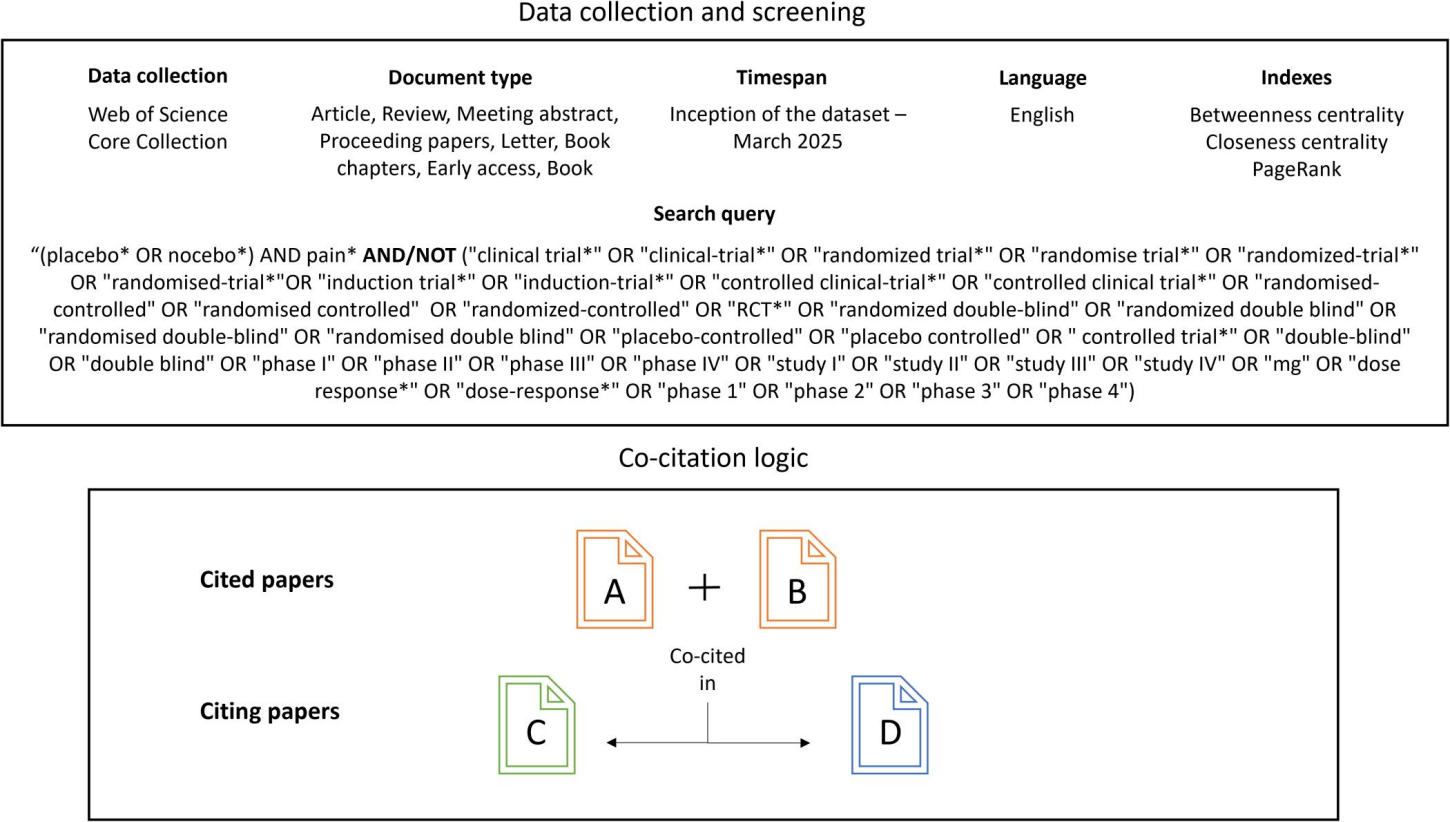
Data collection and screening parameters, and examples of co‐citation. The top part of the figure summarises the parameters used in the scientometric analysis of the literature. The bottom part of the figure is a visual conceptualisation of the logic of co‐citation. The association between document A and document B is considered a co‐citation link because they are cited together in the reference lists of documents C and D.

A summary of the parameters and logic of the analyses is visualised in Figure 1.

## 3 Placebo Mechanisms Results

### 3.1 Navigating the Landscape of Placebos and Pain Research in Neurophysiology and Psychology

The study analysed 1246 records, showing an annual growth rate of 3.97%, pointing to a dynamic and steadily growing area of investigation. The documents, on average, were 9.93 years old (Table 1) and had an average of 47.79 citations, reflecting the scientific relevance and recognition achieved in the literature, supported by a network of 29,296 references. The study found 2954 contributing authors, with 115 having single‐authored documents and an average of 4.33 co‐authors per document. This reflects a collaborative trend, supported by 32.5% of international co‐authorship, showing the integration of diverse scientific perspectives within the field. The breakdown of document types was dominated by 859 articles, highlighting original articles as the primary medium for disseminating research findings, followed by reviews (216), meeting abstracts (60), review book chapters (32), letters (31), and article book chapters (25), underscoring the importance of synthesising, evaluating, and disseminating the existing literature.

**TABLE 1:** General information for the placebo mechanism scientometric.

Description	Results
MAIN INFORMATION ABOUT DATA	
Time span	1985:2025
Sources (Journals, Books, etc)	459
Documents	1246
Annual Growth Rate %	3.97
Average document age	9.93
Average citations per doc	47.79
References	29296
DOCUMENT CONTENTS	
Keywords Plus (ID)	2082
Authors Keywords (DE)	1723
AUTHORS	
Authors	2954
Authors of single‐authored docs	115
AUTHORS COLLABORATION	
Single‐authored docs	142
Co‐Authors per Doc	4.33
International co‐authorships %	32.5
DOCUMENT TYPES	
article	859
article; book chapter	25
article; early access	6
article; proceedings paper	10
letter	31
meeting abstract	60
proceedings paper	6
review	216
review; book chapter	32
review; early access	1

*Note:* The table provides a descriptive overview of the scientific output related to research on pain, placebo, and nocebo mechanisms. It indicates the time period considered, along with the main sources consulted, the documents analysed, and the content covered.

Within the scientometric dataset of placebo and nocebo mechanistic studies, several journals emerged as the main contributors to the field. *PAIN* leads with 131 documents. Following, the *Journal of Pain* records 50 documents, while the *European Journal of Pain* and *Journal of Neuroscience* have 30 documents each (Table 2).

**TABLE 2:** Top‐10 most productive sources.

Sources	Articles
PAIN	131
JOURNAL OF PAIN	50
EUROPEAN JOURNAL OF PAIN	30
JOURNAL OF NEUROSCIENCE	30
FRONTIERS IN PSYCHIATRY	25
FRONTIERS IN PSYCHOLOGY	24
PLOS ONE	24
SCIENTIFIC REPORTS	23
PLACEBO AND PAIN: FROM BENCH TO BEDSIDE	20
JOURNAL OF PSYCHOSOMATIC RESEARCH	19

*Note:* The table lists the main sources contributing to pain and placebo research based on the number of documents published on each source related to the topic.

In terms of *most cited sources* (i.e., citations from the selected dataset), *PAIN* leads with 8075 citations, followed by *Journal of Neuroscience*, with 3085 citations, and *Science*, with 1657. In fourth place, *Neuroimage* stands with 1344, indicating the strive to research the neurobiological correlation of the placebo effect (Table 3).

**TABLE 3:** Top‐10 most cited sources.

Sources	Articles
PAIN	8075
J NEUROSCI	3085
SCIENCE	1657
NEUROIMAGE	1344
NEURON	1126
PLOS ONE	1099
LANCET	1003
P NATL ACAD SCI USA	908
J PAIN	878
JAMA‐J AM MED ASSOC	830

*Note:* The table displays the 10 most cited sources, that is, how many citations a source received from other documents included in the collection as well.

**FIGURE 2:**
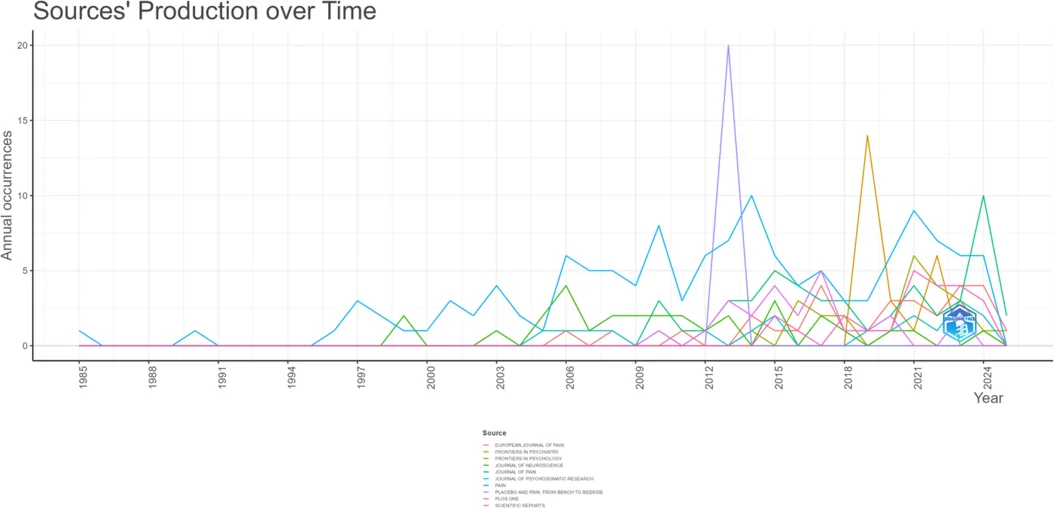
Sources' production over time. The figure shows the sources' production over time, illustrating heterogeneous publication trends across the main journals.

These journals highlight both the clinical and mechanistic focus of placebo research, reflecting its dual anchoring in medical and neuroscience domains. As shown in Figure 2, the trend sources' activity reflects a steady increase over time, with *PAIN* having a higher number of documents compared to other sources over the years, and *Frontiers in Psychiatry* having a high number of documents published between 2018 and 2021. In 2024, the most prolific source for documents on placebo mechanism and pain was the *Journal of Neuroscience*. Interestingly, between 2012 and 2015 the most relevant source for the topic was a book published in 2013, *Placebo and Pain: from bench to bedside*, which probably served as a summary of the field in those prolific years.

In terms of authors who highly contribute to the groth of the field, *Benedetti F* was the most locally cited author (4113 citations), followed by *Colloca L* (2677), *Wager TD* (1527), and *Kirsch I* (1342), reflecting the prominent influence of these authors within placebo mechanism research (Table 4). Table 5 summarises the most prolific authors in placebo mechanism research. *Colloca L* (94 publications) and *Benedetti F* (90 publications) were the leading contributors, followed by *Evers AWM* and *Vase L*. Fractionalised publication counts indicate that these authors maintained high scientific productivity while participating in extensive collaborative research networks.

**TABLE 4:** Top‐10 most locally cited authors.

Author	Local citations
BENEDETTI F	4113
COLLOCA L	2677
WAGER TD	1527
KIRSCH I	1342
AMANZIO M	1281
PRICE DD	1226
KAPTCHUK TJ	1196
ZUBIETA JK	1003
VASE L	928
BINGEL U	893

*Note:* The table displays the 10 authors most cited locally, that is, how many citations an author received, cited by other documents included in the collection.

**TABLE 5:** Top‐10 most prolific authors.

Authors	Articles	Articles fractionalised
COLLOCA L	94	28.57
BENEDETTI F	90	28.84
EVERS AWM	47	8.53
VASE L	45	11.37
KAPTCHUK TJ	39	10.02
BINGEL U	38	8.63
KIRSCH I	36	9.09
WAGER TD	32	9.09
GEERS AL	31	6.61
CARLINO E	29	7.28

*Note:* The table shows the 10 authors with the highest number of published articles in the field of pain and placebo research, reporting both the total number of articles and the fractionalised count, which reflects each author's proportional contribution to co‐authored publications.

**FIGURE 3:**
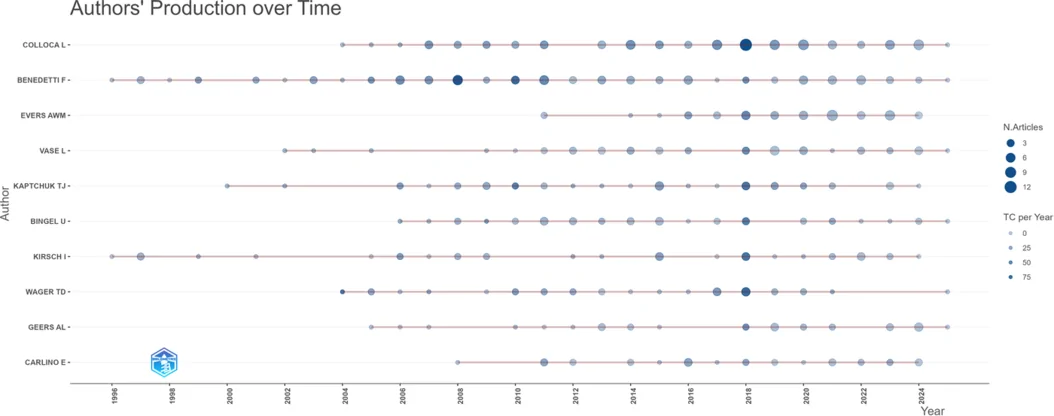
Authors' production over time. The figure shows the authors' production over time represented through circles whose dimensions refer to the number of articles published, while the intensity of the shade refers to the number of citations per year (TC).

In Figure 3, authors' production in terms of documents published and total citations received are presented.

The *most locally cited documents* resulting from the analysis are *Wager TD (2004)* [25] publication in Science with 326 citations, *Amanzio M (1999)* [26] publication in the Journal of Neuroscience obtained 293 citations. *Petrovic P (2002)* [27] Science and *Benedetti F (2003)* [28] in the Journal of Neuroscience follow with 249 and 239 citations each (Table 6).

**TABLE 6:** Top‐10 most locally cited documents.

Document	Local citations
WAGER TD, 2004, SCIENCE ‐ “*Placebo‐Induced Changes in fMRI in the Anticipation and Experience of Pain*”	326
AMANZIO M, 1999, J NEUROSCI ‐ “*Neuropharmacological dissection of placebo analgesia: expectation‐activated opioid systems versus conditioning‐activated specific sub‐systems*”	293
PETROVIC P, 2002, SCIENCE ‐ “*Placebo and opioid analgesia—imaging a shared neuronal network*”	249
BENEDETTI F, 2003, J NEUROSCI ‐ “*Conscious Expectation and Unconscious Conditioning in Analgesic, Motor, and Hormonal Placebo/Nocebo Responses*”	239
PRICE DD, 1999, PAIN ‐ “*Psychological mechanisms of pain and analgesia*”	202
EIPPERT F, 2009, NEURON ‐ “*Activation of the opioidergic descending pain control system underlies placebo analgesia*”	201
ZUBIETA JK, 2005, J NEUROSCI ‐ “*Placebo Effects Mediated by Endogenous Opioid Activity on μ‐Opioid Receptors*”	197
PRICE DD, 2008, ANNU REV PSYCHOL ‐ “*A comprehensive review of the placebo effect: recent advances and current thought*”	190
COLLOCA L, 2008, PAIN ‐ “*Learning potentiates neurophysiological and behavioral placebo analgesic responses*”	187
SCOTT DJ, 2008, ARCH GEN PSYCHIAT ‐ “*Placebo and nocebo effects are defined by opposite opioid and dopaminergic responses*”	183

*Note:* The table presents the top‐10 most locally cited documents, meaning the works that receive the highest number of citations within the selected dataset, thus reflecting their central influence in shaping the specific body of placebo research.

In the context of where the dataset research was carried out, the countries with more documents production are the *USA*, *Germany*, *Italy*, and the *UK* (Table 7).

**TABLE 7:** Top‐10 most productive countries.

Country	Freq
USA	1234
GERMANY	390
ITALY	371
UK	329
NETHERLANDS	298
AUSTRALIA	166
CANADA	147
CHINA	133
DENMARK	121
SWITZERLAND	101

*Note:* The table shows the top‐10 most productive countries, based on the highest number of publications in the dataset.

The *most locally cited countries* are, again, the *USA*, *Italy*, *Germany,* and the *UK* (Table 8), with leading universities being the *University System of Maryland*, the *University of Maryland Baltimore*, *Harvard University,* and *Leiden University* (Table 9).

**TABLE 8:** Top‐10 most locally cited countries.

Country	TC	Average article citations
USA	21964	66.60
ITALY	9453	78.10
GERMANY	7098	52.20
UNITED KINGDOM	3741	43.50
CANADA	2265	45.30
SWEDEN	2084	99.20
NETHERLANDS	2002	33.40
AUSTRALIA	1805	30.60
DENMARK	1500	41.70
NORWAY	1419	43.00

*Note:* The table reports the top‐10 most locally cited countries, that is, those whose publications receive the highest number of citations within the selected dataset, highlighting their leading role and influence in shaping placebo research.

**TABLE 9:** Top‐10 most productive universities.

Affiliation	Articles
UNIVERSITY SYSTEM OF MARYLAND	206
UNIVERSITY OF MARYLAND BALTIMORE	203
HARVARD UNIVERSITY	184
LEIDEN UNIVERSITY	172
UNIVERSITY OF TURIN	155
HARVARD UNIVERSITY MEDICAL AFFILIATES	133
LEIDEN UNIVERSITY ‐ EXCL LUMC	131
AARHUS UNIVERSITY	93
HARVARD MEDICAL SCHOOL	93
LEIDEN UNIVERSITY MEDICAL CENTER (LUMC)	86

*Note:* The table shows the top‐10 most productive affiliations/universities, based on the highest number of publications in the dataset.

#### 3.1.1 A Quantitative Analysis of the *Document* Co‐Citation Network

**FIGURE 4:**
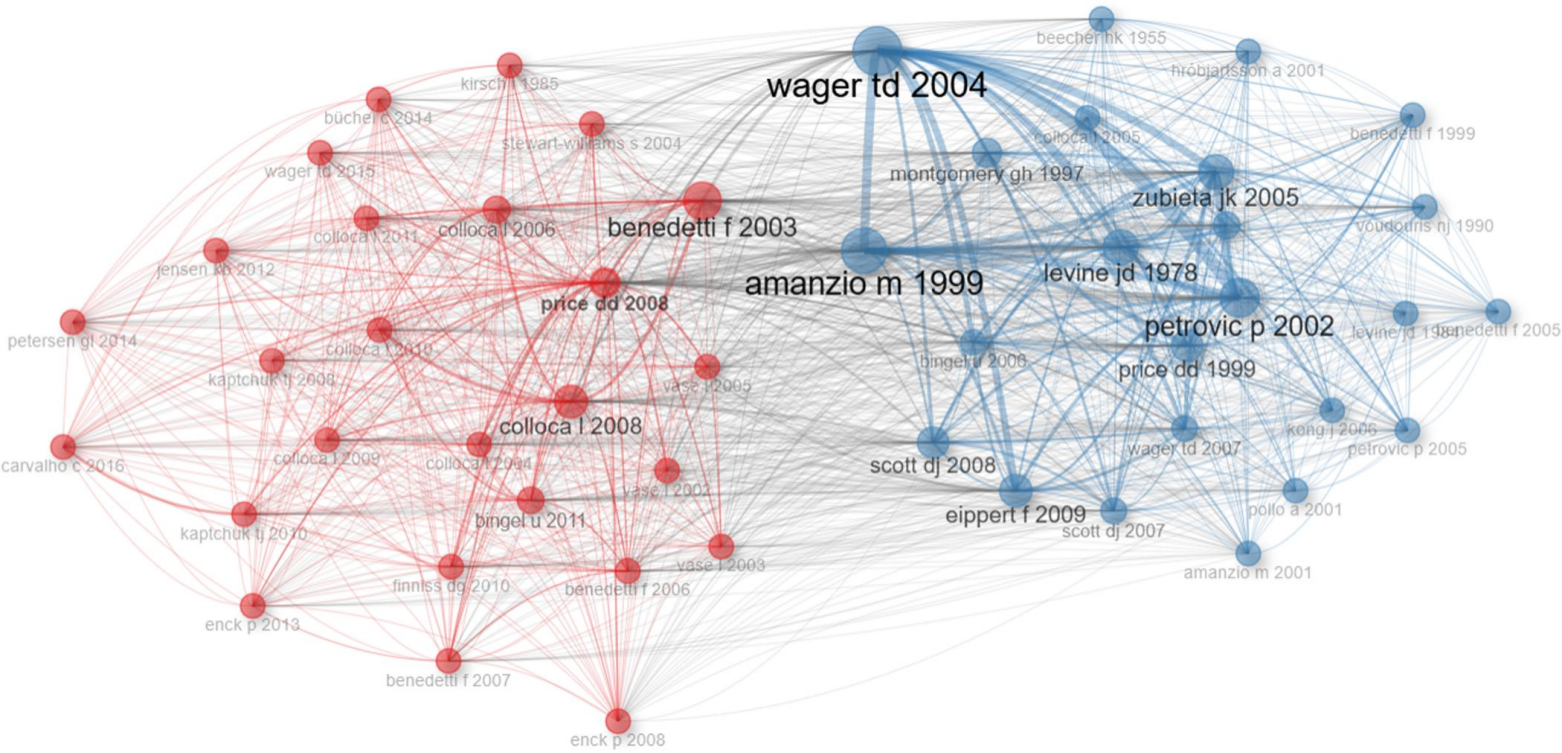
Co‐citation network of documents. The figure displays the results of the co‐citation network analysis of documents, revealing two crucial central works with a high degree of co‐citation. Cluster 1 (red) and Cluster 2 (blue).

The co‐citation network of placebo research identifies two large and interconnected clusters that structure the field (Figure 4). Betweenness centrality confirms the key role of certain works in bridging different domains of placebo studies, while also highlighting the consolidation of both clinical and neurobiological approaches. Other indexes like closeness and PageRank did not yield findings that contributed to our understanding of the network dynamics.

*Cluster 1* (red) represents the clinical, psychological, and conceptual dimensions of placebo effects. The most central document is *Benedetti F (2003)* [28] with a betweenness centrality of 45.82. This work provides one of the first systematic demonstrations of the neurobiological basis of placebo analgesia, identifying the involvement of endogenous opioids and linking clinical observations with neuropharmacological mechanisms. Benedetti's work is followed by *Price DD (2008)* [29] and *Colloca L (2008)* [30] with betweenness centralities of 23.43 and 20.75. The first demonstrated how cognitive factors such as expectations and attention modulate pain perception through top‐down mechanisms, providing a neurocognitive framework for placebo analgesia, while in parallel the latter experimentally disentangled the roles of conditioning and verbal suggestions, showing that placebo responses arise from distinct learning processes that shape clinical outcomes. Other influential nodes include *Vase L (2005)* [31] (17.01), *Colloca L (2006)* [32] (16.06), *Colloca L (2004)* [33] (16.41), and *Benedetti F (2006)* [34] (16.63), which collectively consolidate the clinical neuroscience aspects of placebo. *Cluster 2* (blue) is more mechanistic and neurobiological, anchored by *Wager TD (2004)* [25] with a betweenness centrality of 43.68 and *Amanzio M (1999)* [26] with 39.27. The first demonstrated through neuroimaging that placebo analgesia engages prefrontal and cingulate regions involved in pain modulation, establishing a direct link between expectation and brain activity, while the latter provided one of the earliest pharmacological demonstrations that placebo analgesia is mediated by endogenous opioids, blocked by naloxone. High centrality is also shown by *Eippert F (2009)* [35] and *Scott DJ (2008)* [36], with betweenness centralities of 24.71 and 24.09, both of which used advanced neuroimaging techniques to reveal how a placebo activates descending pain‐control systems and dopaminergic reward pathways. Another key contribution is *Price DD (1999)* [37] (17.65), which provided a psychophysical framework for understanding the multidimensional nature of placebo analgesia. Foundational psychobiological works such as *Levine JD (1978)* [38] (16.14) offered the first clinical demonstration of naloxone‐reversible placebo effects, while *Petrovic P (2002)* [27] (16.86) showed the overlap between opioid analgesia and placebo responses in the brain, further corroborated by *Zubieta JK (2005)* [39] (16.54) and *Montgomery GH (1997)* [40] (19.49). Taken together, the two clusters illustrate how Cluster 1 is more clinically and conceptually oriented, while Cluster 2 grounds placebo in experimental neuroscience. Authors such as Benedetti, Colloca, and Wager act as bridges between these two traditions, ensuring integration across clinical and mechanistic perspectives.

#### 3.1.2 A Quantitative Analysis of the *Keyword* Co‐Occurence Network

**FIGURE 5:**
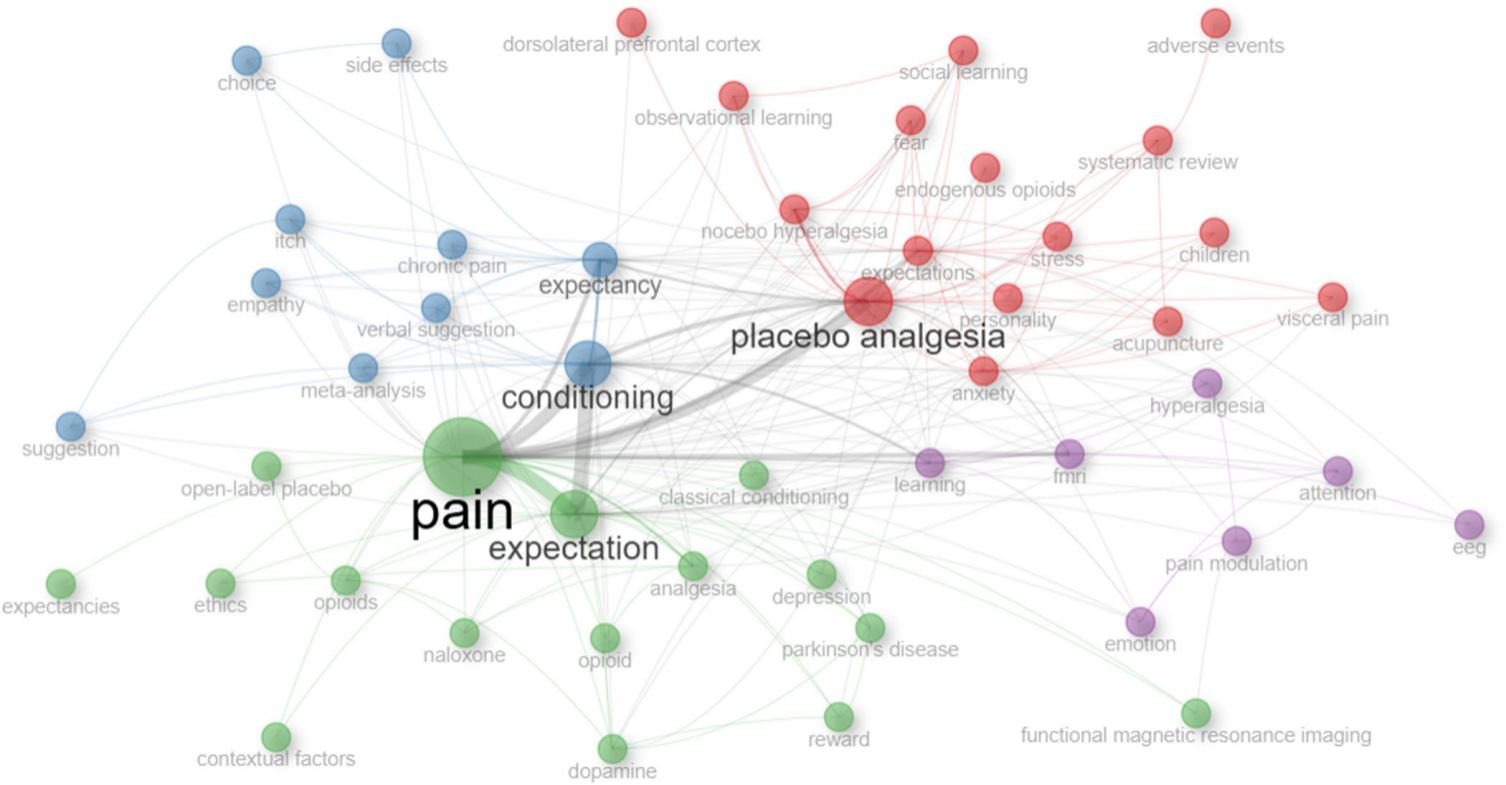
Co‐occurence network of keywords. Nodes represent keywords, and links connect terms that occur together within the same documents. Node size reflects keyword frequency, whereas link thickness indicates the strength of the co‐occurrence relationship. Cluster 1 (red); Cluster 2 (blue); Cluster 3 (green); Cluster 4 (purple). *Note:* the keywords ‘expectation’ and ‘expectancies’ are displayed as separate nodes because they were analysed as indexed in the original publications, without semantic harmonisation. Their separation reflects lexical variation and subtle conceptual differences across the literature rather than entirely distinct research domains.

The keyword co‐occurrence network illustrates how specific terms tend to appear together across the analysed literature, revealing the thematic structure of research in the field (Figure 5). This type of analysis identifies clusters of keywords that frequently co‐occur in the same documents, indicating shared conceptual or methodological ground. The network is organised into four main clusters, each reflecting distinct yet interconnected research domains.

*Cluster 1* (red) is anchored by *placebo analgesia* with the highest betweenness centrality of 102.46, followed by *systematic review* (47.76) and *expectations* (26.58). Additional terms such as *anxiety* (10.99), *nocebo hyperalgesia* (1.25), and *observational learning* (0.39) contribute to this cluster. The coexistence of mechanistic and methodological terms indicates that this cluster bridges laboratory research and evidence‐based medicine, linking expectancy and nocebo paradigms with knowledge synthesis practices such as systematic reviews and meta‐analyses.

*Cluster 2* (blue) is more peripheral but conceptually relevant. It is centred on *conditioning* with a betweenness centrality of 42.90, followed by *expectancy* (19.94). Other nodes include *meta‐analysis* (1.19), *verbal suggestion* (0.32), and *empathy* (0.01). This cluster underscores the importance of learning processes, communication, and relational variables in shaping placebo responses, alongside the growing role of methodological contributions.

*Cluster 3* (green) is the largest and most central cluster of the network. It is dominated by *pain* with a betweenness centrality of 605.78, followed by *expectation* (126.86). Pharmacological terms such as *analgesia* (2.32), *opioids* (2.06), and *dopamine* (1.89) also appear here, together with disease‐related terms such as *Parkinson's disease* (0.46) and *depression* (0.31). This cluster consolidates the central role of pain and its modulation in placebo research, while also highlighting the extension of placebo studies into neurological and psychiatric contexts.

*Cluster 4* (purple), finally, is composed of experimental and methodological terms, with *fMRI* having the highest betweenness centrality of the cluster with 6.12, followed by *learning* with a betweenness of 3.01. Other relevant nodes include *pain modulation* (0.95), *hyperalgesia* (0.63), and *attention* (0.46). This cluster reflects the strong reliance on cognitive neuroscience and psychophysiology, showing how neuroimaging and learning paradigms are used to trace the modulation of pain by expectancy.

Taken together, these clusters show that placebo research is structured around two major anchors—the centrality of *pain* and the conceptual core of *placebo analgesia*—while also expanding into methodological, clinical, and contextual dimensions.

#### 3.1.3 A Quantitative Analysis of the *Collaboration* Network

**FIGURE 6:**
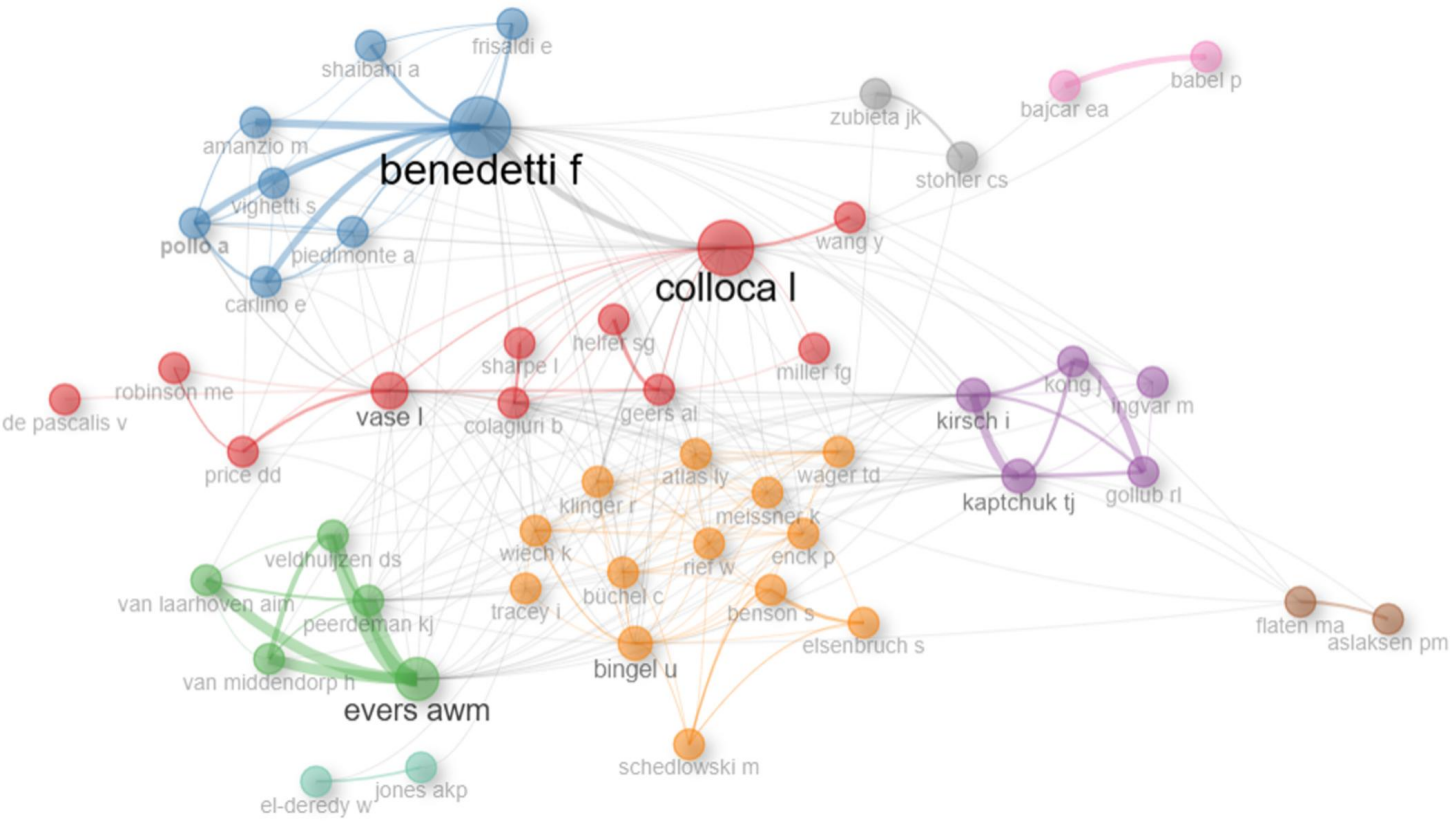
Collaboration network of authors. The figure displays the results of the author collaboration network analysis, revealing the main collaborative communities that have shaped the development of placebo and nocebo research in pain. Cluster 1 (red); Cluster 2 (blue); Cluster 3 (green); Cluster 4 (purple); Cluster 5 (orange); Cluster 6 (brown); Cluster 7 (pink); Cluster 8 (grey); Cluster 9 (light blue).

The author collaboration network illustrates how researchers are connected across the analysed literature, revealing the social structure of placebo mechanism research in pain and the collaborative communities that have driven its development (Figure 6). This type of analysis identifies clusters of authors who have frequently co‐authored publications. This network is organised into nine collaborative communities.

*Cluster 1* (red) is centred on *Colloca L*, who shows the highest betweenness centrality of the cluster (240.10), followed by *Vase L* (130.84), *Geers AL* (49.04), and *Colagiuri B* (25.67). This community has primarily investigated the psychological mechanisms underlying placebo analgesia, demonstrating the role of expectancy, learning, conditioning, and patient‐physician interactions in shaping pain perception and treatment outcomes.

*Cluster 2* (blue) is anchored by *Benedetti F*, who exhibits the highest betweenness centrality of the entire collaboration network (286.32), followed by *Carlino E* (2.37) and *Amanzio M* (0.70). This cluster represents the historical core of placebo mechanisms research, providing seminal evidence on the neurobiological basis of placebo effects and endogenous opioid mechanisms. This group took part in establishing the biological foundation of placebo research, fostering its translation from experimental neuroscience to clinical applications.

*Cluster 3* (green) is led by *Evers AWM* (58.72) and *Peerdeman KJ* (17.27). Their work has mainly focused on psychosocial and contextual determinants of placebo responses, investigating how patient expectations, cognitive factors, and clinical context influence symptom perception and therapeutic outcomes.

*Cluster 4* (purple) is organised around *Kaptchuk TJ* (65.50) and *Kirsch I* (42.48). This community has substantially contributed to the conceptual understanding of placebo effects, integrating expectancy theory, clinical medicine, and neuroimaging evidence while promoting the translation of placebo research into clinical practice.

*Cluster 5* (orange) is centred on *Bingel U* (134.53), *Tracey I* (94.39), *Wager TD* (40.50), *Wiech K* (21.29), and *Enck P* (16.23). This collaborative group is characterised by the extensive use of functional neuroimaging to identify the cortical and subcortical networks underlying placebo analgesia, clarifying how cognitive processes regulate pain through brain mechanisms.

Finally, the remaining smaller collaborative communities include authors such as *Flaten MA* (15.45), *Aslaksen PM, Babel P*, and *Bajcar EA*, representing more specialised lines of research investigating individual differences, cognitive modulation of pain, and placebo and nocebo responsiveness.

Taken together, these collaborative communities demonstrate that placebo mechanism research has evolved through complementary research groups spanning neurobiology, psychology, neuroimaging, and clinical translation. Authors such as Benedetti, Colloca, Bingel, Tracey, Kaptchuk, and Evers emerge as major collaborative hubs, collectively shaping the interdisciplinary development of placebo mechanism research in pain.

## 4 Clinical Trials Results

### 4.1 Navigating the Landscape of Placebos and Pain Research in Clinical Trials

The study analysed 35,134 records, showing an annual growth rate of 3.52% as an indication of a healthy and expanding research field. The documents, on average, were 12.7 years old (Table 10) and had an average of 41.13 citations, testifying the impact within the scholarly domain, supported by a network of 650,949 references. The study found 124,253 contributing authors, with 1239 having single‐authored documents and an average of 6.09 co‐authors per document. Nodes represent keywords, and links connect terms that occur together within the same documents. Node size reflects keyword frequency, whereas link thickness indicates the strength of the co‐occurrence relationship. This reflects a collaborative trend, supported by a 23.76% of international co‐authorship, illustrating the global nature of the research area. The breakdown of document types was dominated by 26,180 articles, highlighting original articles as the primary medium for disseminating research findings, followed by reviews (6701), proceedings papers, letters, and meeting abstracts, underscoring the importance of synthesising, evaluating, and disseminating the existing literature.

**TABLE 10:** General information for the clinical trials scientometric.

Description	Results
MAIN INFORMATION ABOUT DATA	
Time span	1985:2025
Sources (Journals, Books, etc)	3650
Documents	35,134
Annual Growth Rate %	3.52
Document Average Age	12.7
Average citations per doc	41.13
References	650,949
DOCUMENT CONTENTS	
Keywords Plus (ID)	30,622
Author's Keywords (DE)	32,611
AUTHORS	
Authors	124,253
Authors of single‐authored docs	1239
AUTHORS COLLABORATION	
Single‐authored docs	1566
Co‐Authors per Doc	6.09
International co‐authorships %	23.76
DOCUMENT TYPES	
Article	26,180
Article; book	1
Article; book chapter	95
Article; early access	103
Article; proceedings paper	1030
Article; publication with expression of concern	2
Book review	1
Letter	215
Meeting abstract	645
Proceedings paper	107
Reprint	2
Review	6701
Review; book chapter	14
Review; early access	37
Review; publication with expression of concern	1

*Note:* The table provides a descriptive overview of the scientific output related to research on pain, placebo, and nocebo used in clinical trials. It indicates the time period considered, along with the main sources consulted, the documents analysed, and the content covered.

In the examination of the scientometric dataset for the clinical trials, certain journals (sources) emerge as significant contributors to RCTs on pain controlled with placebo arms. The *Cochrane Database of Systematic Reviews* leads with 1190 documents. Following, *PAIN* records 777 documents, while *Anaesthesia and Analgesia* and *Trials* have 489 and 319 documents, respectively (Table 11).

**TABLE 11:** Top‐10 most productive sources.

Sources	Articles
COCHRANE DATABASE OF SYSTEMATIC REVIEWS	1190
PAIN	777
ANAESTHESIA AND ANALGESIA	489
TRIALS	319
PAIN MEDICINE	294
CLINICAL JOURNAL OF PAIN	293
CLINICAL THERAPEUTICS	291
HEADACHE	288
BRITISH JOURNAL OF ANAESTHESIA	276
PLOS ONE	267

*Note:* The table lists the main sources contributing to pain, and RCTs based on the number of documents published on each source related to the topic.

In terms of *most cited sources* (i.e., citations from the selected dataset), *PAIN* leads with 50,655 citations, indicating its central role in the academic discourse and its contribution to pain research and clinical trials. Following, *Anaesthesia and Analgesia* ranks second with 19,189 citations, and third is the *New England Journal of Medicine*, with 18,049 citations. *The Lancet* follows closely with 17,459 citations. These journals represent global leaders in publishing medical research, frequently directing clinical practice and policy (Table 12).

**TABLE 12:** Top‐10 most cited sources.

Sources	Articles
PAIN	50,655
ANESTH ANALG	19,189
NEW ENGL J MED	18,049
LANCET	17,459
COCHRANE DB SYST REV	14,596
ANAESTHESIOLOGY	14,071
NEUROLOGY	13,655
JAMA‐J AM MED ASSOC	12,566
J RHEUMATOL	12,407
GASTROENTEROLOGY	12,368

*Note:* The table displays the 10 most cited sources, that is, how many citations a source received from other documents included in the collection as well.

**FIGURE 7:**
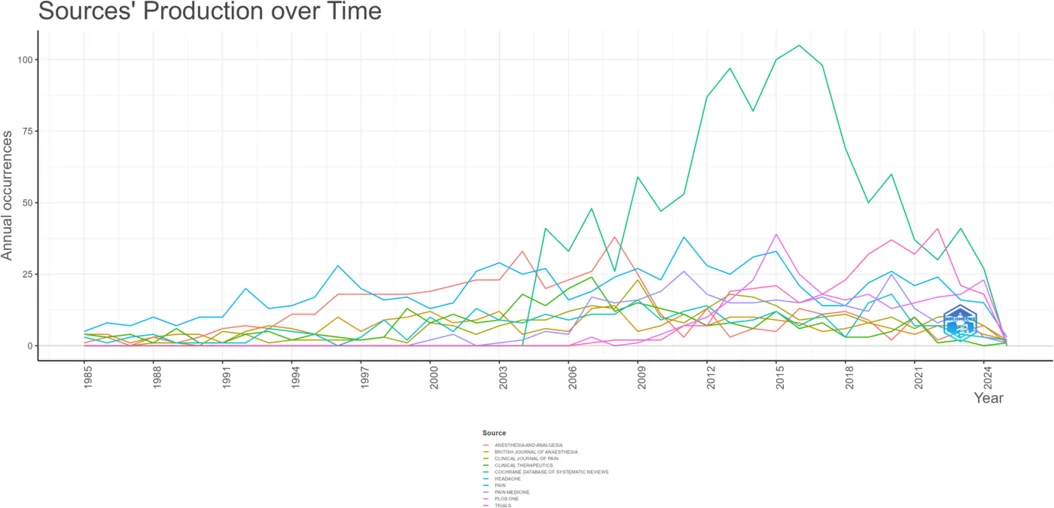
Sources' production over time. The figure shows the sources' production over time, illustrating a marked difference between the first five top‐ranked journals.

As shown in Figure 7, the trend in sources' activity reflects a steady increase over time, with the *Cochrane Database of Systematic Reviews* having more than twice the number of documents compared to other sources between 2012 and 2018, probably highlighting the need to consolidate the existing literature and to guide the standardisation of practices within the discipline.

The most locally cited authors are summarised in Table 13. *Manchikanti L* ranked first with 5715 local citations, followed by *Moore RA* (5269), *Cash KA* (4902), and *Pampati V* (4582). These findings identify the authors whose publications have had the greatest influence within the selected clinical trial dataset, reflecting their central role in the development of placebo‐controlled pain research. Additionally, Table 14 summarises the most prolific authors in placebo‐controlled clinical trial research. *Moore RA* (221 publications) was the leading contributor, followed by *Derry S* (167), *McQuay HJ* (105), *Drewes AM* (100), and *Zhang Y* (98). Fractionalised publication counts indicate that these authors maintained high scientific productivity while participating in extensive collaborative research networks.

**TABLE 13:** Top‐10 most locally cited authors.

Author	Local citations
MANCHIKANTI L	5715
MOORE RA	5269
CASH KA	4902
PAMPATI V	4582
MCQUAY HJ	3527
DERRY S	3055
JENSEN TS	2607
DWORKIN RH	2238
SINGH V	2190
LAMOREAUX L	2008

*Note*: The table displays the 10 authors most cited locally, that is, how many citations an author received, cited by other documents included in the collection.

**TABLE 14:** Top‐10 most prolific authors.

Authors	Articles	Articles fractionalised
MOORE RA	221	55.15
DERRY S	167	43.26
MCQUAY HJ	105	26.40
DREWES AM	100	16.18
ZHANG Y	98	14.57
WANG Y	92	13.99
LIPTON RB	91	14.62
BUCHBINDER R	88	16.11
ARENDT‐NIELSEN L	87	16.34
KEHLET H	87	19.14

*Note:* The table shows the 10 authors with the highest number of published articles in the field of pain and RCTs, reporting both the total number of articles and the fractionalised count, which reflects each author's proportional contribution to co‐authored publications.

**FIGURE 8:**
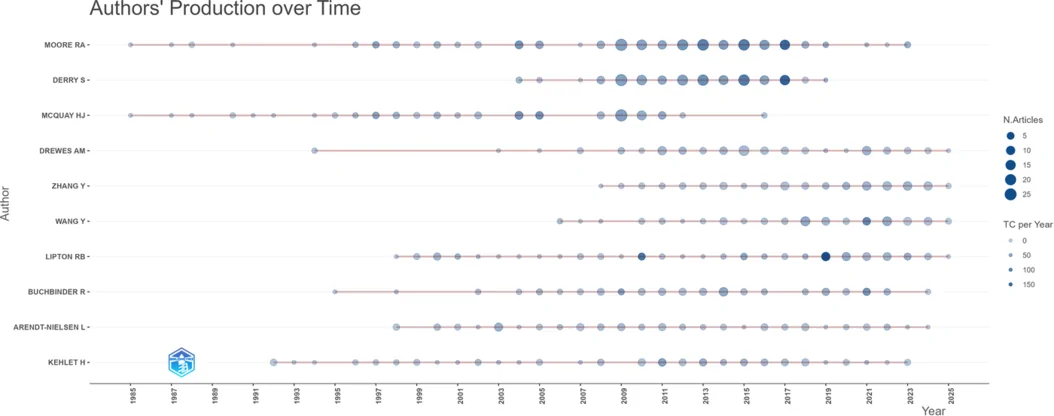
Authors' production over time. The figure shows the authors' production over time represented through circles whose dimensions refer to the number of articles published, while the colour refers to the number of citations per year (TC).

In Figure 8, authors' production in terms of documents published and total citations received are presented.

The most locally cited documents resulting in the analysis. *Farrar JT (2001)* [41] publication on PAIN is the most cited with 737 citations. *Backonja M (1998)* [42] and *Rowbotham M (1998)* [43] both published on JAMA‐Journal of the American Medical Association obtained 322 citations and 304 respectively. *Goldstein DJ (2005)* [44] published on PAIN comes in fourth with 295 citations, *MANCHIKANTI L (2008)* with 269 [45], *ARNOLD LM (2004)* with 254 [46], *MAX MB (1992)* with 252 [47], *ROSENSTOCK J* (2004) with 245 [48], *MOORE RA (1998)* with 240 [49], and *DWORKIN RH (2003)* with 240 [50] (Table 15).

**TABLE 15:** Top‐10 most locally cited documents

Document	Local citations
FARRAR JT, 2001, PAIN ‐ "*Clinical Importance of Changes in Chronic Pain Intensity Measured on an 11‐Point Numerical Pain Rating Scale*"	737
BACKONJA M, 1998, JAMA‐J AM MED ASSOC ‐ "*Gabapentin for the Symptomatic Treatment of Painful Neuropathy in Patients with Diabetes Mellitus: A Randomized Controlled Trial*"	322
ROWBOTHAM M, 1998, JAMA‐J AM MED ASSOC ‐ "*Gabapentin for the Treatment of Postherpetic Neuralgia: A Randomized Controlled Trial*"	304
GOLDSTEIN DJ, 2005, PAIN ‐ "*Duloxetine versus Placebo in Patients with Painful Diabetic Neuropathy*"	295
MANCHIKANTI L, 2008, PAIN PHYSICIAN ‐ "*Comprehensive evidence‐based guidelines for interventional techniques in the management of chronic spinal pain*"	269
ARNOLD LM, 2004, ARTHRITIS RHEUM‐US ‐ "*A Double‐Blind, Multicenter Trial Comparing Duloxetine with Placebo in the Treatment of Fibromyalgia Patients with or without Major Depressive Disorder"*	254
MAX MB, 1992, NEW ENGL J MED. ‐ *Effects of desipramine, amitriptyline, and fluoxetine on pain in diabetic neuropathy*	252
ROSENSTOCK J, 2004, PAIN ‐ *Pregabalin for the treatment of painful diabetic peripheral neuropathy: a double‐blind, placebo‐controlled trial*	245
MOORE RA, 1998, PAIN ‐ *Size is everything‐‐large amounts of information are needed to overcome random effects in estimating direction and magnitude of treatment effects*	269240
DWORKIN RH, 2003, NEUROLOGY ‐ *Pregabalin for the treatment of postherpetic neuralgia: a randomized, placebo‐controlled trial*	240

*Note:* The table presents the top‐10 most locally cited documents, meaning the works that receive the highest number of citations within the selected dataset, thus reflecting their central influence in shaping the specific body of placebo research.

In the context of where the dataset research was carried out, the countries with more documents production are the *USA*, *China*, the *UK*, and *Canada* (Table 16).

**TABLE 16:** Top‐10 most productive countries.

Country	Freq
USA	38,384
CHINA	9475
UK	9130
CANADA	6726
GERMANY	5690
AUSTRALIA	5100
ITALY	4904
IRAN	4410
FRANCE	4290
DENMARK	3536

*Note:* The table shows the top‐10 most productive countries, based on the highest number of publications in the dataset.

The most cited countries are the *USA*, *United Kingdom*, *Canada,* and *Germany* (Table 17), with leading universities being *Harvard University*, *University of California System*, *Harvard University of Medical Affiliates*, and the *University of Toronto* (Table 18).

**TABLE 17:** Top‐10 most cited countries.

Country	TC	Average article citations
USA	558,241	57.00
UNITED KINGDOM	124,629	52.70
CANADA	75,378	52.70
GERMANY	70,960	47.70
AUSTRALIA	51,698	43.90
FRANCE	48,477	61.10
CHINA	47,896	18.30
DENMARK	43,824	47.40
NETHERLANDS	42,274	52.00
ITALY	40,872	33.00

*Note:* The table reports the top‐10 most cited countries, that is, those whose publications receive the highest number of citations within the selected dataset, highlighting their leading role and influence in shaping placebo research.

**TABLE 18:** Top‐10 most productive universities.

Affiliation	Articles
HARVARD UNIVERSITY	2018
UNIVERSITY OF CALIFORNIA SYSTEM	1703
HARVARD UNIVERSITY MEDICAL AFFILIATES	1521
UNIVERSITY OF TORONTO	1492
UNIVERSITY OF COPENHAGEN	1406
UNIVERSITY OF LONDON	1068
PFIZER	990
ASSISTANCE PUBLIQUE HOPITAUX PARIS (APHP)	930
HARVARD MEDICAL SCHOOL	884
MAYO CLINIC	866

*Note:* The table shows the top‐10 most productive affiliations/universities, based on the highest number of publications in the dataset.

#### 4.1.1 A Quantitative Analysis of the *Document* Co‐Citation Network

**FIGURE 9:**
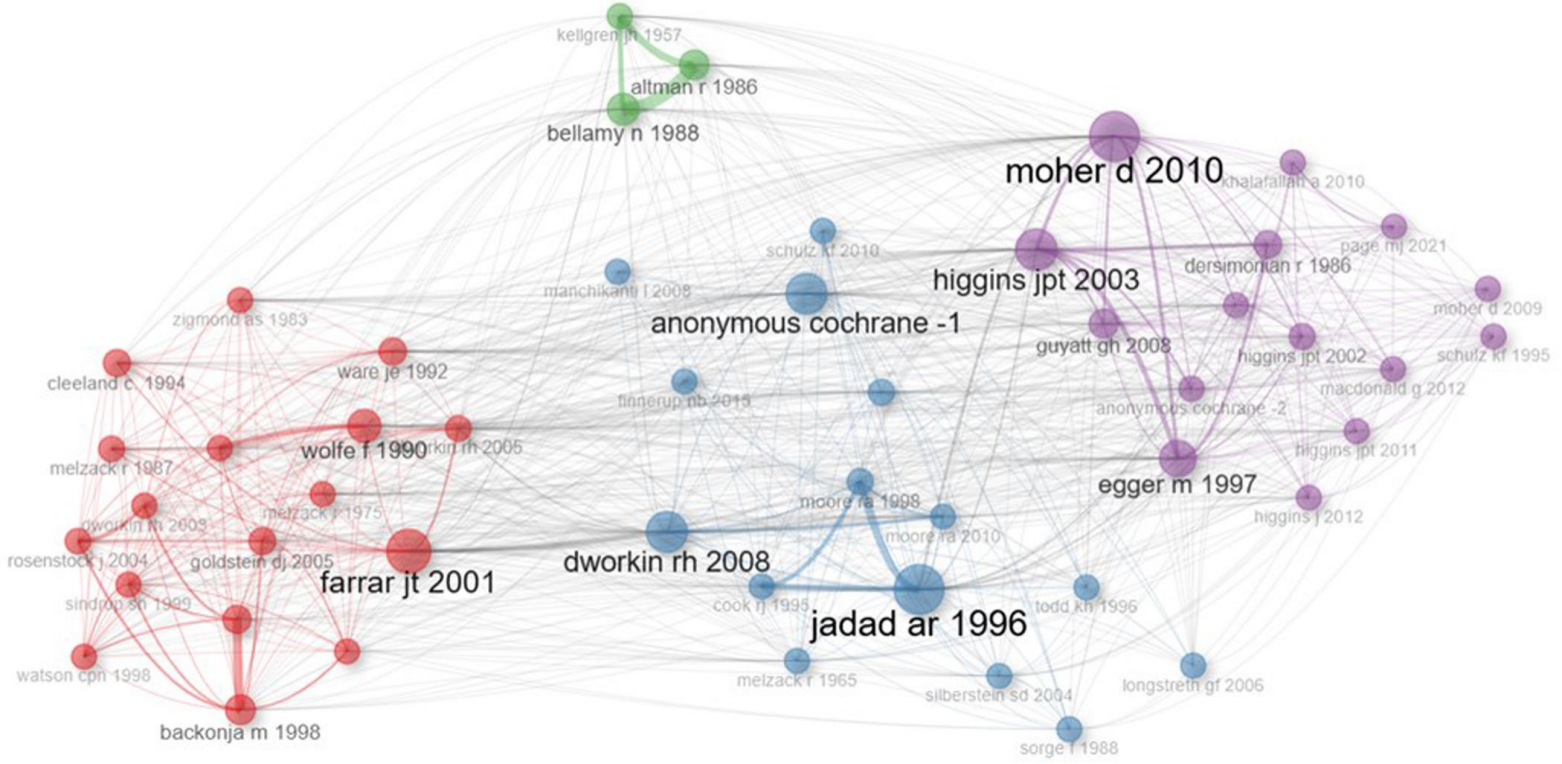
Co‐citation network of documents. The figure displays the results of the co‐citation network analysis of documents, revealing several crucial central works with a high degree of co‐citation. Cluster 1 (red); Cluster 2 (blue); Cluster 3 (green); Cluster 4 (purple).

The co‐citation network of documents reveals several crucial central works with a high degree of co‐citation (Figure 9). This demonstrates the significant contribution of many researchers in carrying out RCTs. The analysis considers betweenness centrality as the only metric that provides insightful results, while other indices like closeness and PageRank did not yield findings that contributed to our understanding of the network dynamics.

Our results highlight four central clusters. *Cluster 1* (red), with *Farrar JT (2001)* [41] having the highest betweenness centrality of the cluster with 55.17, followed by *Dworkin RH (2005)* [51] (21.30). These documents have a foundational role in the understanding of a significant clinical reduction of pain intensity and in the standardised outcomes in pain clinical trials.

In *Cluster 2* (blue), two documents predominantly facilitate information flow across their cluster and with other parts of the network, holding the highest indexes of betweenness centrality of the network: *Jadad AR (1996)* [52] (108.84) and *Dworkin RH (2008)* [53] (107.79). These documents introduce, respectively, a widely used scale to assess the methodological rigour of RCTs and the updated recommendations for outcomes in chronic pain trials.

*Cluster 3* (green) appears more peripheral within the network, characterised by low betweenness centrality and limited connection to core clusters, suggesting a more independent line of research. Its leading document is *Bellamy N (1988)* [54], with a betweenness centrality of 9.74, it specifically focuses on the standardisation of pain and function monitoring in patients with osteoarthritis.

*Cluster 4* (purple) holds a structurally central position within the network, driven by two highly influential methodological papers: *Moher D (2010)* [55] and *Higgins JPT (2003)* [56]. These documents provide essential methodological guidance for conducting and reporting systematic reviews and meta‐analyses. The first introduces the PRISMA statement, a widely adopted reporting guideline, while the latter presents the I^2^ statistic, a key tool for measuring heterogeneity in meta‐analyses.

#### 4.1.2 A Quantitative Analysis of the *Keyword* Co‐Occurence Network

**FIGURE 10:**
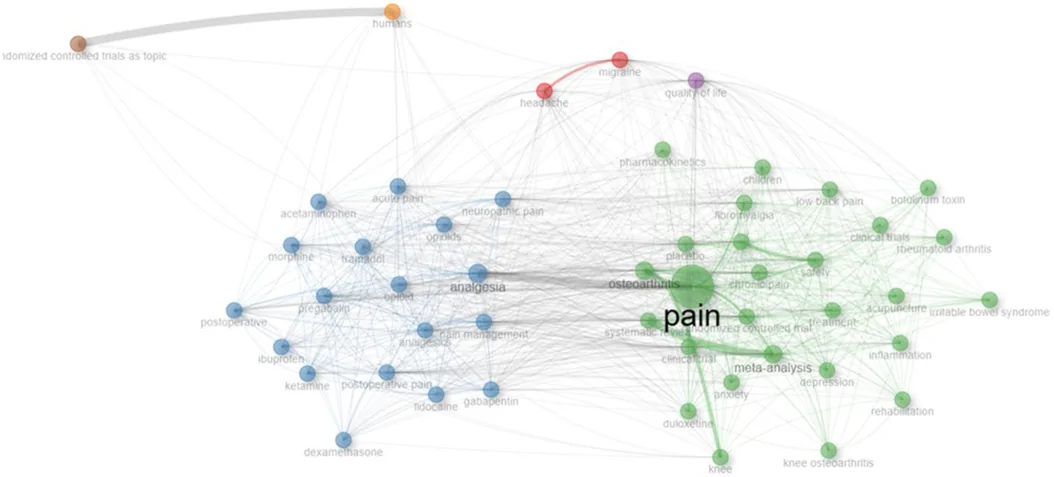
Co‐occurence network of keywords. The figure displays the results of the keyword co‐occurrence analysis. Nodes represent keywords, and links connect terms that occur together within the same documents. Node size reflects keyword frequency, whereas link thickness represents co‐occurrence strength. Cluster 1 (red); Cluster 2 (blue); Cluster 3 (green); Cluster 4 (purple); Cluster 5 (orange); Cluster 6 (brown).

The keyword co‐occurrence network illustrates how specific terms tend to appear together across the analysed literature, revealing the thematic structure of research in the field (Figure 10). This type of analysis identifies clusters of keywords that frequently co‐occur in the same documents, indicating shared conceptual or methodological ground. The network is organised into six main clusters, each reflecting distinct yet interconnected research domains.

*Cluster 1* (red) is a small cluster centred on primary headache disorders, including terms such as *migraine* and *headache,* with betweenness centrality of 2.38 and 0.78. These keywords reflect a focused research interest in these conditions, possibly in relation to treatment approaches and classification. *Cluster 2* (blue) includes a dense grouping of keywords related to postoperative and pharmacological pain management, keywords which predominantly link the cluster are, among others *analgesia* (15.99), *pain management* (8.12), *analgesics* (5.86), *postoperative pain* (4.57), and *morphine* (3.09). This cluster indicates a strong biomedical focus on drug‐based interventions and acute pain contexts. *Cluster 3* (green) is the most extensive and central cluster in the network. It encompasses broad, foundational terms in our dataset, such as *pain*, with a betweenness centrality of 239,08, followed by *meta‐analysis* (21.44), *osteoarthritis* (12.65), *systematic review* (10.92), *randomised controlled trial* (922), *chronic pain* (8.60), *placebo* (6.21), and *clinical trial* (4.68). These keywords underscore the importance of evidence‐based research methodology and pain conceptualisation across conditions. Notably, terms like treatment, safety, fibromyalgia, and low back pain also fall within this cluster, highlighting key clinical applications. *Cluster 4* (purple) focuses on *quality of life*, which emerges as a distinct but connected thematic node, suggesting its transversal relevance across clinical outcomes, with a betweenness centrality of 12.49. *Cluster 5* (orange) includes more general terms like *humans*, which likely reflect results from indexing keyword practices, with a betweenness centrality of 3.64. *Cluster 6* (brown), finally, appears peripheral, containing only the keyword randomised controlled trials as topic, possibly reflecting articles more focused on methodological discussions than specific clinical issues, with a low betweenness centrality of 0.002.

#### 4.1.3 A Quantitative Analysis of the Collaboration Network

**FIGURE 11:**
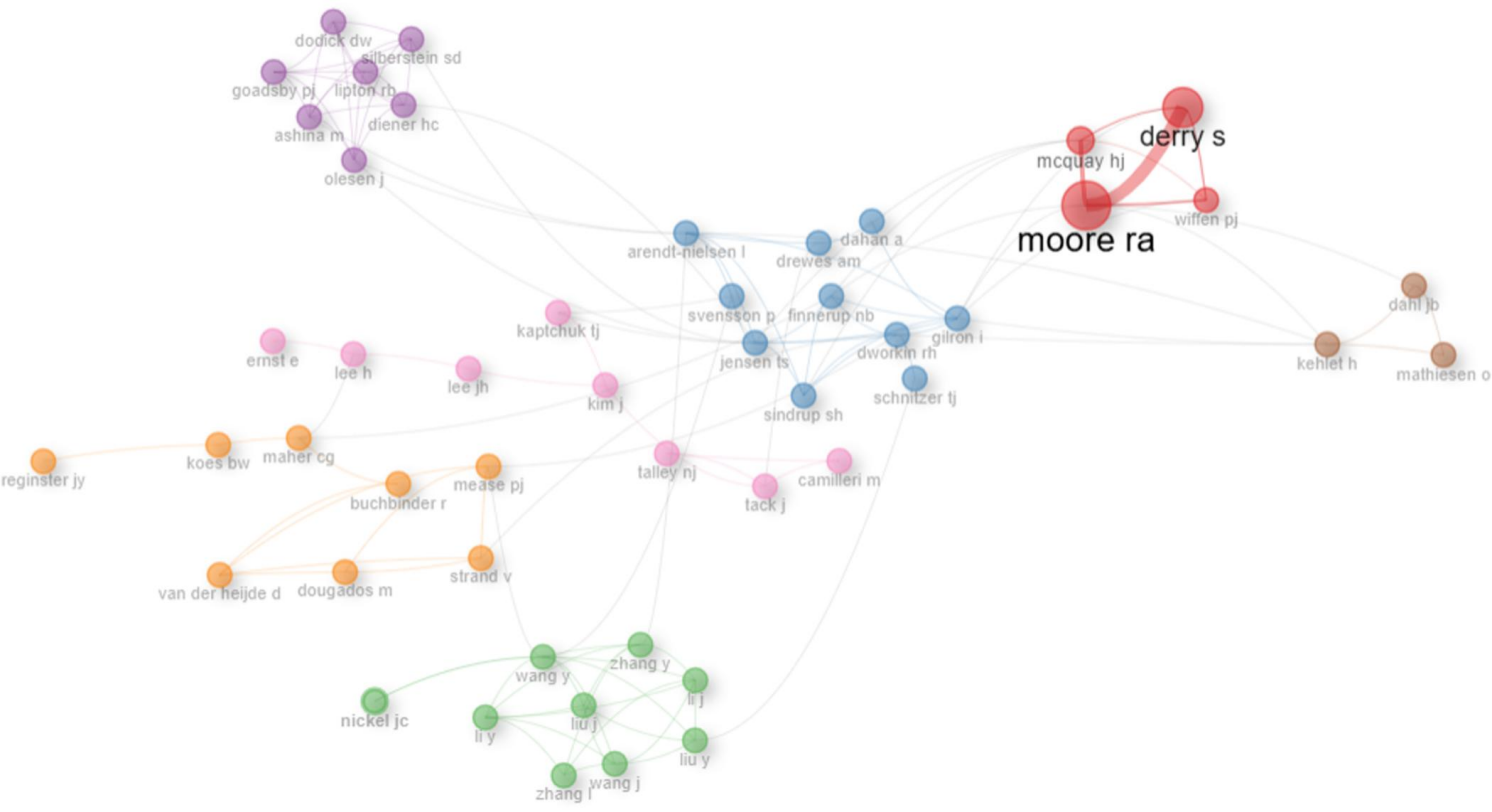
Collaboration network of authors. The figure displays the results of the author collaboration network analysis, revealing the principal collaborative communities and their relationships within the field. Cluster 1 (red); Cluster 2 (blue); Cluster 3 (green); Cluster 4 (purple); Cluster 5 (orange); Cluster 6 (brown); Cluster 7 (pink).

The author collaboration network illustrates how researchers are connected across the analysed literature, revealing the collaborative structure underlying clinical trials involving placebo in pain research (Figure 11). This type of analysis identifies clusters of authors who have frequently co‐authored publications. This network is organised into seven collaborative communities.

*Cluster 1* (red) is centred on *Moore RA*, who shows the highest betweenness centrality of the cluster (37.17), followed by *McQuay HJ* (33.97). Other contributors include *Derry S* (0.14) and *Wiffen PJ* (0.14). This community has substantially contributed to evidence synthesis and pharmacological pain management, producing systematic reviews and meta‐analyses that have shaped evidence‐based recommendations for analgesic therapies.

*Cluster 2* (blue) is anchored by *Arendt‐Nielsen L*, who exhibits the highest betweenness centrality of the entire collaboration network (321.32), followed by *Jensen TS* (283.36), *Finnerup NB* (184.64), *Dworkin RH* (175.73), *Drewes AM* (126.91), and *Gilron I* (123.51). This collaborative group focuses on neuropathic pain, quantitative sensory testing, and clinical trial methodology, contributing to the development of standardised outcome measures and improving the understanding and treatment of chronic pain conditions.

*Cluster 3* (green) is led by *Wang Y* (154.97) and *Zhang Y* (134.80), together with *Li Y, Liu Y*, and colleagues. This community primarily reflects large‐scale clinical research and randomised controlled trials evaluating pharmacological interventions for pain, contributing to the rapid expansion of evidence from Asian research centres.

*Cluster 4* (purple) is organised around *Olesen J* (46.52), *Ashina M* (37.81), *Silberstein SD* (36.17), and *Diener HC* (32.71). This group has mainly investigated headache and migraine disorders, contributing to the evaluation of therapeutic strategies and outcome measures in placebo‐controlled clinical trials.

*Cluster 5* (orange) is centred on *Maher CG* (203.01), followed by *Mease PJ* (99.28), *Koes BW* (47.00), *van der Heijde D* (41.21), and *Buchbinder R* (33.60). This collaborative community focuses on musculoskeletal disorders, including low back pain, osteoarthritis, and rheumatic diseases, providing high‐quality clinical evidence to guide conservative and pharmacological treatments.

*Cluster 6* (brown) is led by *Kehlet H* (71.45) together with *Dahl JB* (6.98), representing a specialised line of research on postoperative pain management and perioperative analgesia. Finally, *Cluster 7* (pink) is organised around *Tack J* (100.97), *Lee H* (76.87), *Kaptchuk TJ* (58.88), and *Kim J* (58.14), integrating placebo mechanisms with functional gastrointestinal disorders and emphasising the translation of placebo research into clinical practice.

Taken together, these collaborative communities demonstrate that clinical trial research has evolved through specialised groups addressing different pain conditions and methodological approaches. Authors such as Arendt‐Nielsen, Jensen, Moore, Maher, Kehlet, and Tack emerge as major collaborative hubs, collectively advancing evidence‐based pain management and the methodological standards of placebo‐controlled clinical trials.

## 5 Discussion

This scientometric analysis provides a comprehensive overview of placebo and nocebo research within the field of pain, mapping its evolution, identifying central actors, and capturing emerging themes. Previous bibliometric efforts have been limited in scope: the 2016 analysis relied on a small set of terms and returned just over 300 articles until November 2011 [28], while the 2022 study was restricted to the JIPS database and presented a selective snapshot of the field [29]. By contrast, our dataset offers a more integrative perspective, combining mechanistic and clinical approaches. This allows us to appreciate the dual role of placebo in pain research: on the one hand as an object of neurobiological and psychological investigation, on the other hand as a methodological challenge and tool in randomised controlled trials (RCTs).

A central finding is the persistent asymmetry between the two domains. Despite decades of research elucidating the biological and psychological processes underlying placebo responses, the overwhelming majority of documents originate from RCTs, where the placebo serves as a control condition rather than as the primary object of investigation. This paradox highlights that although placebo is one of the most frequently cited concepts in the medical literature, it is far more often invoked as a methodological device than studied as a phenomenon in its own right.

Both domains show strong but distinct publication landscapes. Journals such as PAIN and Journal of Neuroscience are central for mechanistic studies, while clinical trial publications are dominated by Cochrane Database of Systematic Reviews and Anaesthesia and Analgesia. The authorship networks reflect parallel trends: Benedetti, Colloca, and Wager stand out in mechanistic research, while Moore and Manchikanti dominate the clinical side. Geographically, Italy, Germany, and the USA lead mechanistic placebo studies, while the USA, China, and the UK dominate RCT production, suggesting that mechanistic research is concentrated in a few specialised groups, while clinical trial research is more widely distributed.

Document co‐citation networks reinforce this division while pointing to bridges across domains. Mechanistic clusters are anchored by seminal works such as Wager et al. (2004) and Amanzio and Benedetti (1999), which established the biological and psychological foundations of placebo analgesia through neuroimaging and psychopharmacological experiments. The RCT network is structured around methodological landmarks such as Jadad et al. (1996) and Dworkin et al. (2008), which defined standards for trial quality and outcome measures. Key figures such as Benedetti and Colloca act as bridges linking both traditions.

The keyword co‐occurrence analysis reveals the thematic organisation of the field. In mechanistic studies, terms such as pain, expectation, and conditioning dominate, while dopamine, opioids, and fMRI reflect the centrality of neuroscience. Emerging clusters also highlight increasing attention to relational dimensions including ethics and doctor‐patient communication, indicating a broadening toward clinical and societal implications. In clinical trials, the network is more methodologically oriented, with central terms like randomised controlled trial, systematic review, and meta‐analysis, linked to conditions such as fibromyalgia and low back pain. The convergence of both domains around terms such as pain, analgesia, and systematic review suggests an emerging integration between mechanisms and applications.

The author collaboration network highlights that mechanistic placebo research has developed through a limited number of highly interconnected communities integrating expertise in neurobiology, psychology, neuroimaging, and clinical translation. This collaborative structure reflects the interdisciplinary nature of placebo mechanism research and the central role of a few leading research groups in advancing the field. In contrast, the collaboration network of clinical trials is organised around larger and more specialised communities focused on specific pain conditions and methodological expertise, including neuropathic pain, musculoskeletal disorders, headache, and perioperative analgesia. This organisation reflects the broader clinical scope and multicenter nature of placebo‐controlled trials. Together with the document co‐citation and keyword co‐occurrence analyses, the collaboration networks provide a complementary perspective on the field, showing not only how knowledge has evolved but also how it has been generated through collaborative research communities. These findings further emphasise the distinct yet complementary roles of mechanistic studies and randomised clinical trials in advancing placebo and nocebo research.

However, these findings must be considered in light of some limitations. The dataset was restricted to pain‐related studies, excluding other relevant placebo research on depression, sleep, immune function, and motor performance. In addition, our search strategy prioritised specificity by focusing on studies explicitly using placebo and nocebo terminology. Consequently, studies investigating related mechanisms, such as expectations, conditioning, and contextual effects, without explicitly referring to placebo or nocebo phenomena may have been underrepresented. On top of this, placebo and nocebo studies were analysed together, without examining trends in the two research areas separately. Future scientometric studies should investigate whether placebo and nocebo literature follows different trends and forms different networks. Scientometric analyses remain descriptive, revealing connectivity and influence but not quality or validity directly. The reliance on local citations also limits insight into broader interdisciplinary influence, and recent developments such as digital health applications may not yet appear prominently in the data.

Similar caveats apply to the clinical trial analysis. The large number of RCTs introduces heterogeneity in design, reporting standards, and outcome measures. The strong dominance of systematic reviews and meta‐analyses means that secondary analyses are highly represented, potentially inflating the centrality of evidence synthesis at the expense of primary trial diversity.

In conclusion, this scientometric mapping reveals that placebo and nocebo research in pain has evolved into a dynamic field bridging neuroscience, psychology, and clinical trial methodology, demonstrating both consolidation around key figures and diversification into new topics. Compared to earlier bibliometric efforts, this study offers a broader and more integrated view, situating placebo at the intersection of mechanistic understanding and clinical practice—a dual role with implications for both basic science and therapeutic application.

## Ethics Statement

This study is a scientometric analysis based exclusively on bibliographic records and metadata obtained from previously published literature and publicly accessible scientific databases. No human participants, animals, human biological material, or identifiable personal data were directly involved in this research. Therefore, ethics committee approval and informed consent were not required.

## Conflicts of Interest

The authors declare no conflicts of interest.

## Supporting information


Supporting File

## Data Availability

The datasets generated and/or analysed during the current study are available from the corresponding author upon reasonable request.
