# Supplementary material for: A Comprehensive Scientometric Analysis of Placebo and Nocebo Effects in Pain Research: From Placebo Mechanisms to Clinical Trials
Source: J Eval Clin Pract. 2026 Sep 13;32(6):e70570. doi: 10.1111/jep.70570 (PMC13571793; doi:10.1111/jep.70570)

PRISMA 2020 flow diagram for new systematic reviews which included searches of databases and registers only

Supplementary materials

Table 1A. Screening and Selection of Clinical Trials Records

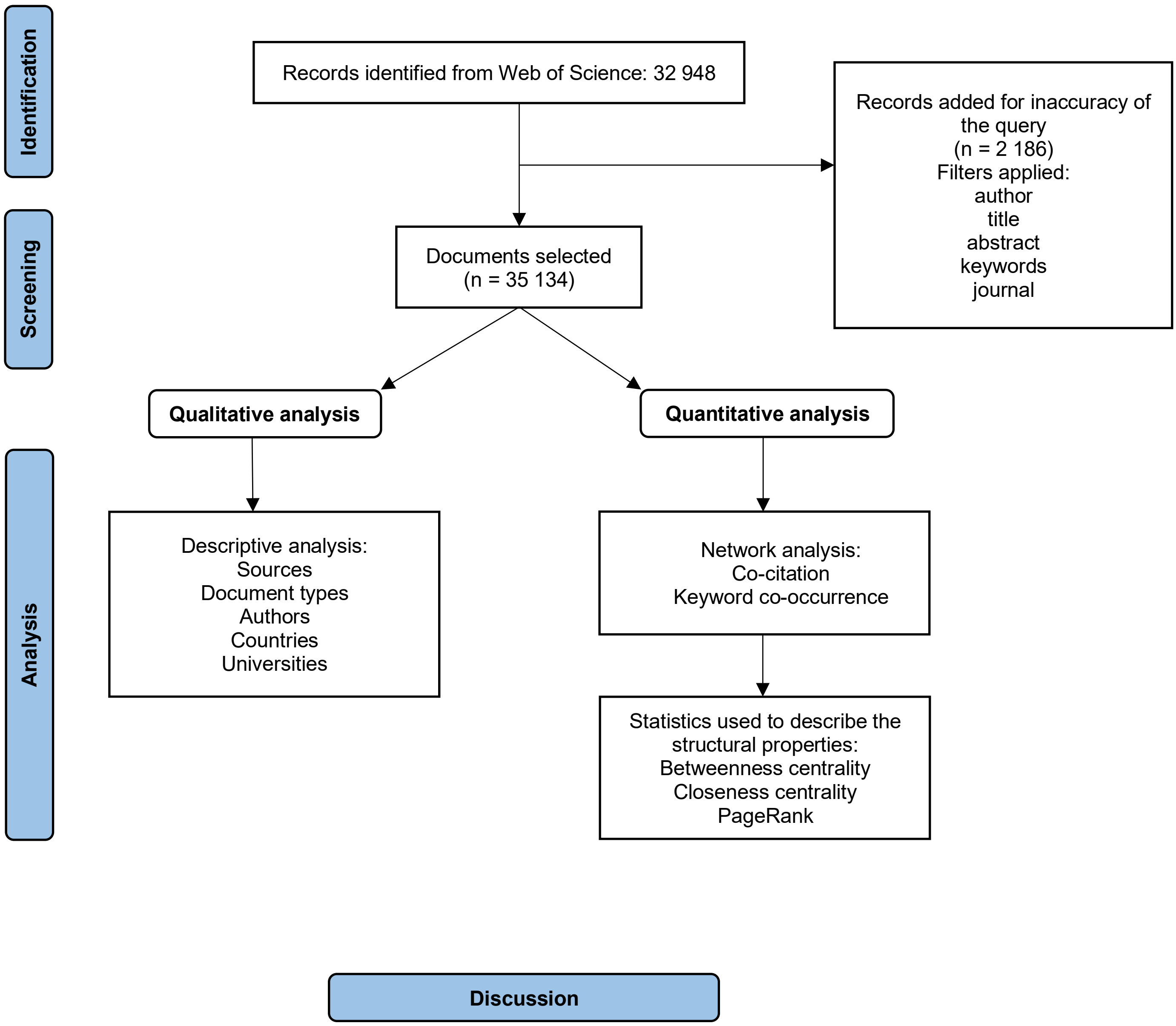

PRISMA 2020 flow diagram for new systematic reviews which included searches of databases and registers only

Table 1B Screening and Selection of Placebo Mechanisms Records

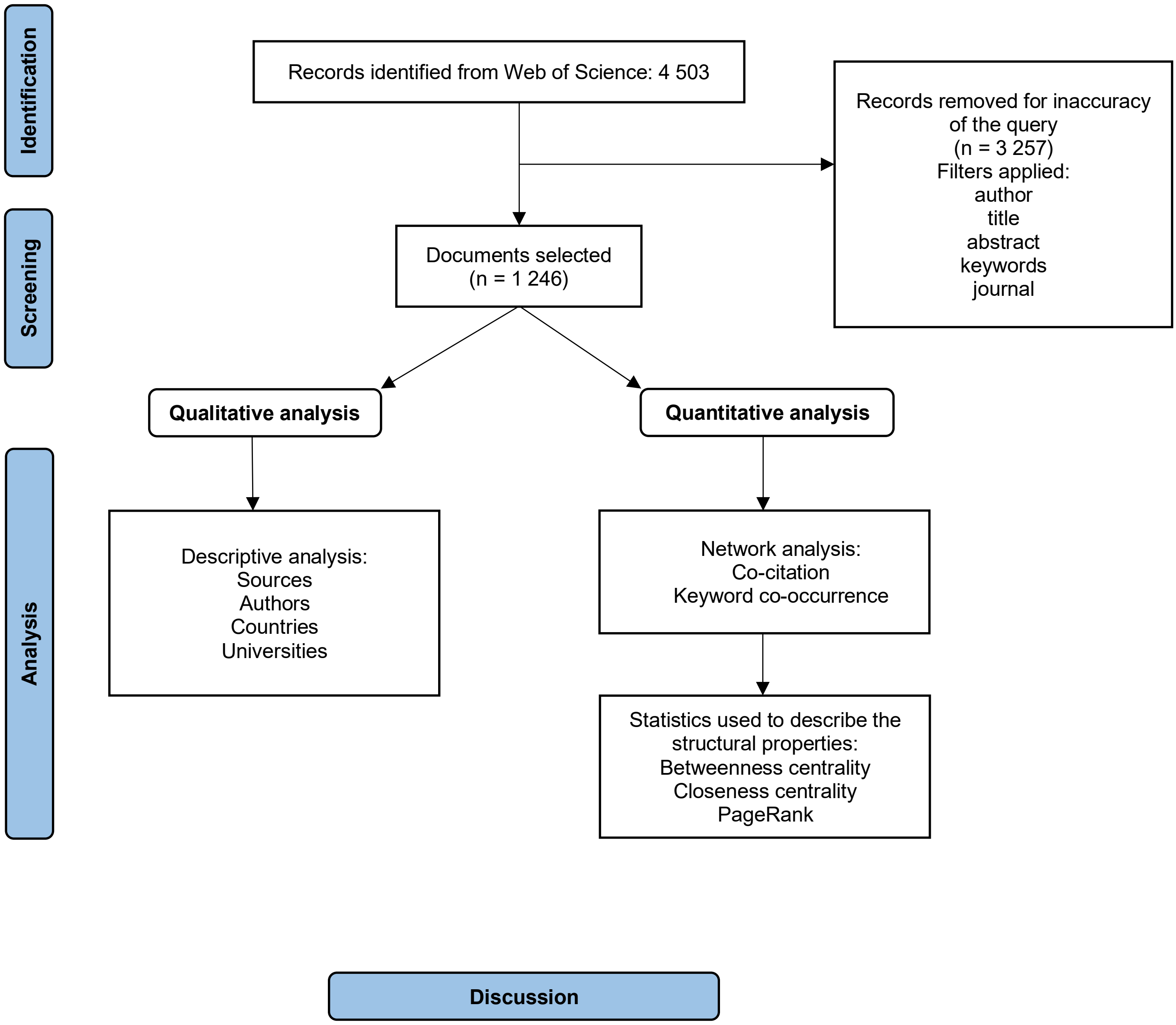

Supplement: Supplementary file 1 [file JEP-32-0-s001.pdf]
